# Optically Controlled Supercapacitors: Functional Active Carbon Electrodes with Semiconductor Particles

**DOI:** 10.3390/ma14154183

**Published:** 2021-07-27

**Authors:** Haim Grebel

**Affiliations:** The Center for Energy Efficiency, Resilience and Innovation (CEERI), The Electronic Imaging Center (EIC), The New Jersey Institute of Technology (NJIT), Newark, NJ 07102, USA; grebel@njit.edu

**Keywords:** functionalized active carbon materials, optical effects in supercapacitors, thermal effects, nano, energy storage

## Abstract

Supercapacitors, S-C—capacitors that take advantage of the large capacitance at the interface between an electrode and an electrolyte—have found many short-term energy applications. The parallel plate cells were made of two transparent electrodes (ITO), each covered with a semiconductor-embedded, active carbon (A-C) layer. While A-C appears black, it is not an ideal blackbody absorber that absorbs all spectral light indiscriminately. In addition to a relatively flat optical absorption background, A-C exhibits two distinct absorption bands: in the near-infrared (near-IR and in the blue. The first may be attributed to absorption by the OH^−^ group and the latter, by scattering, possibly through surface plasmons at the pore/electrolyte interface. Here, optical and thermal effects of sub-μm SiC particles that are embedded in A-C electrodes, are presented. Similar to nano-Si particles, SiC exhibits blue band absorption, but it is less likely to oxidize. Using Charge-Discharge (CD) experiments, the relative optically related capacitance increase may be as large as ~34% (68% when the illuminated area is taken into account). Capacitance increase was noted as the illuminated samples became hotter. This thermal effect amounts to <20% of the overall relative capacitance change using CD experiments. The thermal effect was quite large when the SiC particles were replaced by CdSe/ZnS quantum dots; for the latter, the thermal effect was 35% compared to 10% for the optical effect. When analyzing the optical effect one may consider two processes: ionization of the semiconductor particles and charge displacement under the cell’s terminals—a dipole effect. A model suggests that the capacitance increase is related to an optically induced dipole effect.

## 1. Introduction

Supercapacitors, S-C [1,2,3,4,5,6], either symmetric (the two cell’s electrodes are of the same type), or asymmetric (made with two types of electrodes) [7,8,9], are capacitors that take advantage of the capacitance at the interface between an electrode and an electrolyte. They have found many energy applications due to their fast charging and discharging capabilities. Applications have also been proposed for the future digital micro-grid [10,11] and optical modulators [12]. Though much effort has been invested in the material, chemistry and electrical properties of batteries [13], and of S-C [14,15,16], surprisingly little research has been devoted to optoelectronic effects in S-C [17]. Here, carbon-based, optically controlled S-C that exhibit electrical double-layer behavior are described. The intent here is to gain a basic understanding of the optical and related thermal effects when incorporating SiC particles in active carbon (A-C) based electrodes with a poly(methyl methacrylate) (PMMA) binder and aqueous electrolyte (1 M Na_2_SO_4_). A substantial capacitance increase is demonstrated upon illuminating the cells with a modest light intensity, which is attributed to a polarization effect by the light-activated particles. The paper is organized as follows: In Section 2, descriptions are given for Materials and Methods; Results and Discussions are provided in Section 3 including characterizations, optical and thermal effects; Conclusions are provided in Section 4.

## 2. Materials and Methods

**Figure 1:**
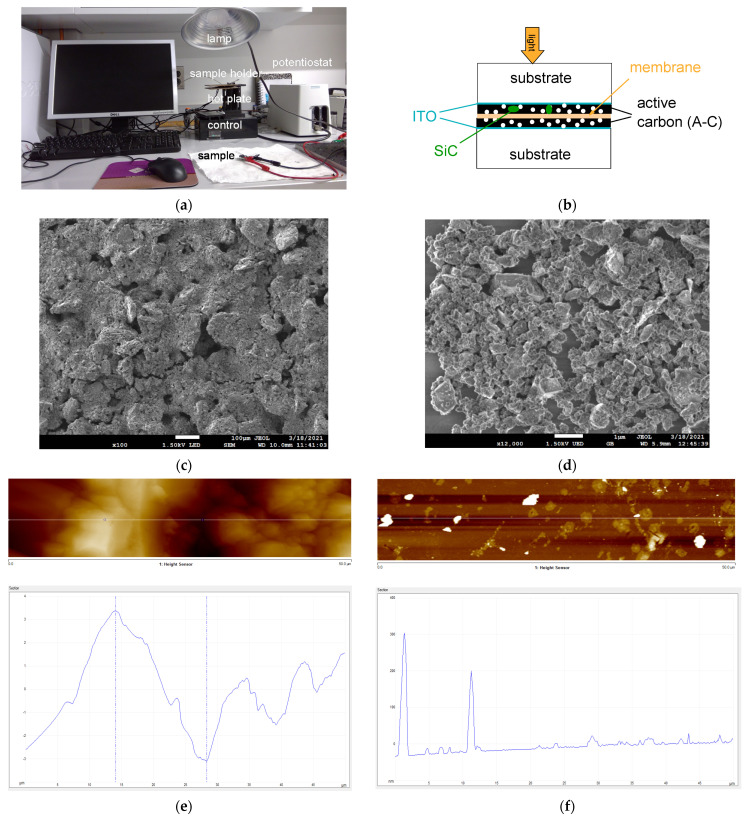
(**a**) Picture of the experimental set-up. (**b**) Schematics of the sample: TS80 is the hydrophilic membrane; ITO: indium-tin-oxide; (**c**) SEM picture of a film composed of A-C with a PMMA binder. The bar is 100 μm. (**d**) SEM picture of sub-micron SiC particles on glass. The bar is 1 μm. (**e**) AFM profile of a film made of A-C with a PMMA binder. (**f**) AFM profile of SiC particles on glass.

The measurement system is pictured in Figure 1a. The basic cell is composed of two transparent electrodes. Glass substrates coated with indium tin oxide (ITO, average sheet resistance R_sqr_ = 6 Ώ and average thickness of ca 220 nm, were made by Huanyu (Yueqing, China). They were facing each other to form a parallel plate capacitor (Figure 1b). The active carbon (A-C) material was produced by General Carbon Company, GCC (Paterson, NJ, USA). Scanning Electron Microscopy (SEM, Jeol, Tokyo, Japan) and Atomic Force Microscopy (AFM, Bruker, Billerica, MA, USA) were used to assess the surface of the exposed and coated electrode on ITO just before cell assembly (Figure 1c–f).

A-C in a PMMA binder with SiC particles: These are typically made with a 100 mg/mL A-C, which was produced by GCC, and a 10 mg/mL PMMA. The poly(methyl methacrylate), PMMA powder (Polyscience, Niles, IL, USA) was first dissolved in toluene at 90 °C for 30 min on a hot plate. The 10 mg/mL of SiC particles (Johnson Metthey, Devens, MA, USA) were then added to the solution and the batch was sonicated for 1 h using a horn-antenna sonicator. Lastly, the A-C was added and mixed with the other components for 2 h. The slurry, drop casted on the ITO coated, glass substrate was let dry out in an oven at 90 °C for 30 min. A typical composite A-C film was ~100 μm thick. The procedure for films embedded with CdSe/ZnS quantum dots followed that of the SiC particles.

Electrolyte: 1 M of Na_2_SO_4_; hydrophilic nano-filtration polyamide filter (TS80, Sterlitech, Kent, WA, USA) used as a separator.

Electrochemical Measurements: Measurements were carried out with a Potentiostat/Galvanostat (Metrohm, Herisau, Switzerland). The samples were illuminated by a 75 W incandescent light bulb situated 30 cm or 62.5 cm above the samples; by varying the distance of the lamp, the light intensity on the cell has changed too. The light intensity of the entire radiation spectra (from the visible to the IR) was measured with a bolometer and was assessed as 30 mW/cm^2^ and 3 mW/cm^2^, respectively. Note that the light intensity at the sample surface did not follow an inverse square law for the distance because the light source is extended not a point source. While using here the simplest of light sources, other illuminators, such as a microscope illuminator are possible provided that their spectral output has a blue-green component. A calibrated homemade hot plate, which was interfaced with a thermocouple was used for the thermal experiments. A second thermocouple assessed the temperature right at the sample surface.

Optical transmission measurements: A computer-controlled monochromator (SPEX, Florham Park, NJ, USA), which was interfaced with a white light source, a chopper, and a Si detector was used to assess the optical transmission of each film when deposited on a glass substrate. The transmission value is defined as the signal obtained with the film on a glass slide divided by the signal obtained with only the glass slide. Each transmission curve was normalized to its peak transmission due to the heterogeneous nature of the drop-casted film (see Figure 1). Of interest are the spectral trends rather than the actual film’s losses. The films used for optical transmission purposes were prepared from a diluted version of the electrode slurry and were thinner, of the order of 10 μm since a ~100 μm film for the S-C was too thick to let the light through.

In order to assess the effect of electrolytes on the optical transmission, the transmissions of wet and dry samples were measured. The sample was first soaked in 0.1 M Na_2_SO_4_ for a couple of hours and its optical absorption was assessed. Then the sample was dried up in an oven for three days at 90 °C.

## 3. Results and Discussions

### 3.1. Characterizations

Imaging via SEM and AFM: Pictures of the surface morphology are shown in Figure 1c–f. Figure 1c shows an SEM picture of A-C with a PMMA binder. It is composed of relatively large ‘chunks’ made of domains. Figure 1d exhibits a large dispersion of SiC with mostly sub-micron particles. AFM picture shows that the lateral domain size of A-C is of the order of 15 μm (Figure 1e) whereas that of SiC is indeed of a sub-micron level (Figure 1f).

**Figure 2:**
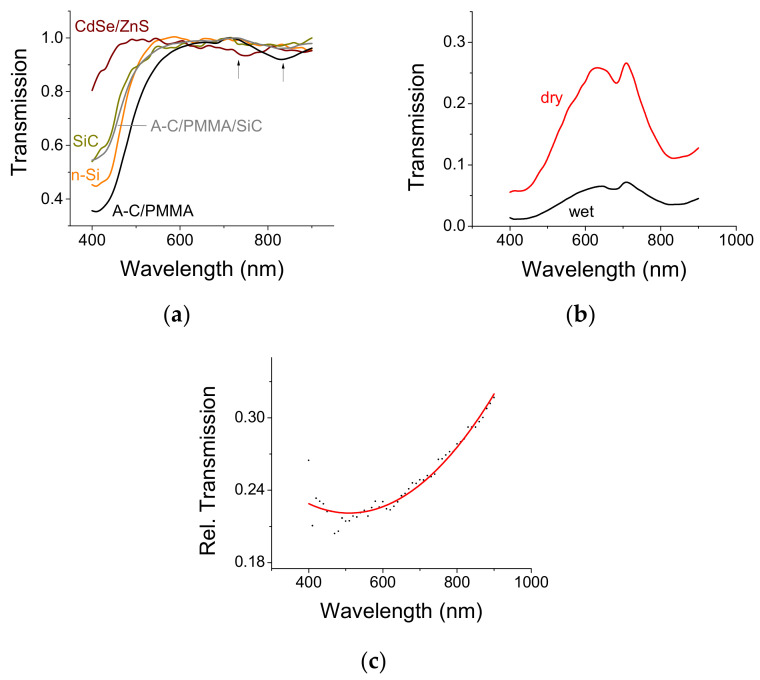
(**a**) Normalized optical transmission through the various film components used in the experiments. The peak transmission was set to 1 for each curve in order to accentuate the spectral response. (**b**) The absorption of A-C with a PMMA binder on a glass slide under dry and wet conditions. The curves are given as-is without peak normalization. (**c**) The relative transmission when the transmission under wet conditions was normalized by the transmission under dry conditions.

Optical Transmission: Optical transmission through the various material components is shown in Figure 2. Each transmission curve was first referenced to its substrate (a glass slide) to remove the effect of glass (if any). Then, the curve was normalized to its peak transmission; as mentioned earlier, the interest is the spectral dependence rather than the particular film loss. The glass slide signal was referenced to the transmission through the air. The transmission of a glass slide is fairly constant throughout the spectral range between 400 to 900 nm. The transmission of the ITO film on glass is flat throughout the visible with a small absorption near the blue region of 400 nm [18]; PMMA has also a flat transmission in the visible with a transmission coefficient of 0.9 [19]. These data have been independently verified but will not be shown here.

The green/yellow alpha SiC powder absorbs heavily in the blue as are n-Si and A-C (see Figure 2a). The response of A-C with a PMMA binder to wet and dry conditions is shown in Figure 2b. The transmission is given without peak normalization because it is the same sample that underwent various treatments. The sample was first soaked in 1 M Na_2_SO_4_ for a couple of hours and its optical absorption was assessed. Then the sample was dried up in an oven for three days at 90 °C. One may observe that the general absorption pattern was retained. There are three major absorption peaks (transmission dips) at 430, 670, and 840 nm, respectively. The latter is attributed to an OH^−^ group because it is accentuated by the presence of water. The middle is attributed to the glass substrate. The more interesting one is at 430 nm.

The absorption curve for water is minimal in the blue and maximal in the red [20]. That means that the optical transmission through water or electrolyte should be maximal in the blue and minimal in the red. If we assume that the film absorption did not change under dry or wet conditions, then by dividing the transmission of the film under wet conditions by the transmission of the film under dry conditions one should obtain the transmission of only the electrolyte (water). This is not the case here; the transmission trend is quite the opposite, namely, it is minimal in the blue/green and maximal in the red. The maximum absorption (the largest dip) is in the green wavelength range. This means that optical scattering is affected by the A-C/electrolyte interface. Such scattering is susceptible to the refractive index of the pore filler (namely, electrolyte or water in our case). The pores within the matrix of A-C are typically of submicron scale and, therefore, may be viewed as inverse Mie scatterers [21,22]. It is proposed that this unusual scattering effect is the result of coupling between the π–π* bonds at the interface of the A-C pores [23] and the electrolyte in the pore.

### 3.2. Optical Related Effects

**Figure 3:**
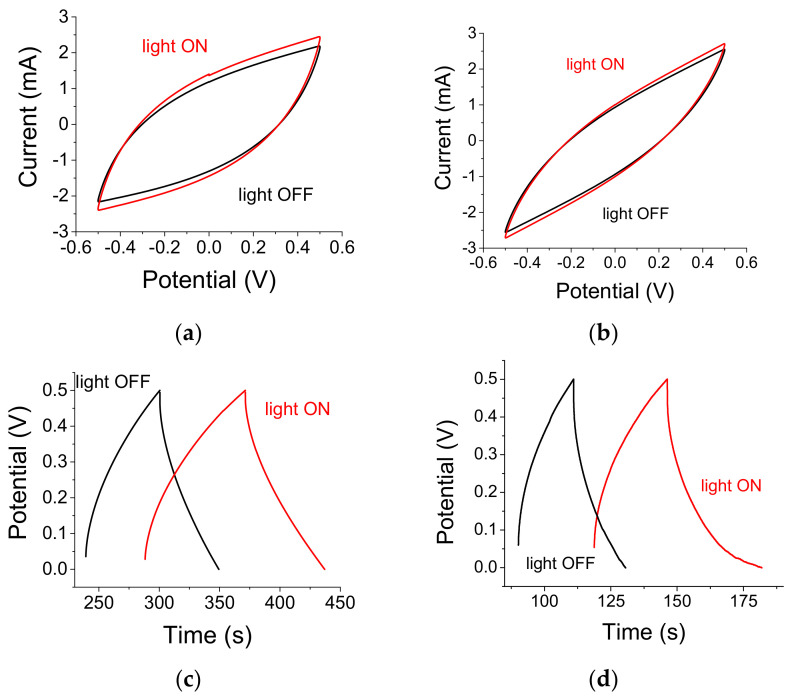
(**a**) CV (at a rate of 0.1 V/s) when a sample is illuminated from the front (where the SiC particles are) and (**b**) when another sample is illuminated from the back (without SiC particles) (**c**) CD for sample (**a**) (excited with a current of 0.2 mA) under light and dark conditions. (**d**) SiC embedded A-C electrode with ITO (front, facing the light) and Al (at the sample back) both used as current collectors.

The capacitance change of the SiC embedded, A-C-based cell is described below. Capacitance increase can be observed in Figure 3a,c. The relative capacitance increase is normalized by its respective illuminated (or non-illuminated) areas. Specifically: ΔC/C = [(C/A)_illum_ − (C/A)_dark_]/(C/A)_dark_, where (C/A)_illum_ is the capacitance measured under illumination and normalized by the light-exposed area; (C/A)_dark_ is the capacitance measured under dark conditions and normalized by the area under dark (typically, the entire capacitor area). Cyclic Voltammetry (CV) indicates a ΔC/C = 27% relative increase whereas the Charge Discharge measurements (CD) alludes to ΔC/C ≈ 60%. Note that the current levels are larger when using CV. CV curves exhibit a tilt towards larger current values, as well as a broadening of the curve waist. A curve tilt without waist broadening is typically associated with sample heating as shown below. Figure 3b shows the optical effect even in the absence of SiC particles; it amounts to ΔC/C ≈ 14% (including the effect of smaller exposed area) and is attributed to the A-C absorption in the blue. An asymmetric cell with two types of current collectors is shown in Figure 3d. While the electrodes were both made of SiC embedded A-C, the current collectors were made of, respectively, ITO and Al. Illumination by the white light source was made through the front ITO film.

**Figure 4:**
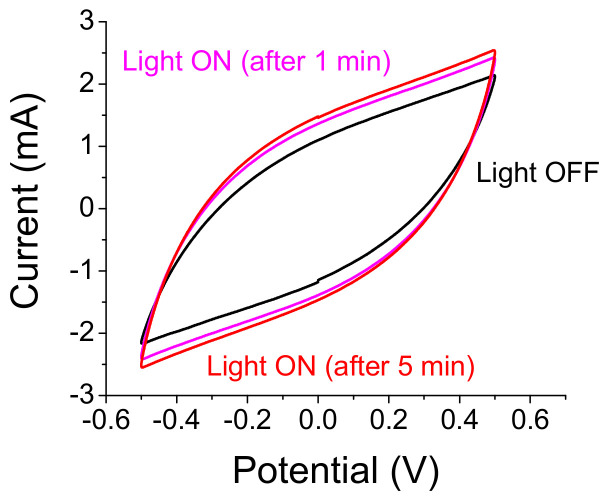
Temporal response of n-Si embedded A-C based S-C. Most of the capacitance increase of 50% occurs within the first min of exposure while a fuller increase of 68% occurs after 5 min.

The time response of the optically controlled capacitors is yet to be found. The first step is to assess the capacitance increase as a function of the illumination time. In Figure 4 we show a capacitance increase of ≈50% for n-Si embedded, A-C electrode cell after 1 min and a further, smaller increase after 5 min of light exposure amounting to a total increase of 68%. Included in the assessment was the smaller exposure area of one-half of the sample surface.

**Figure 5:**
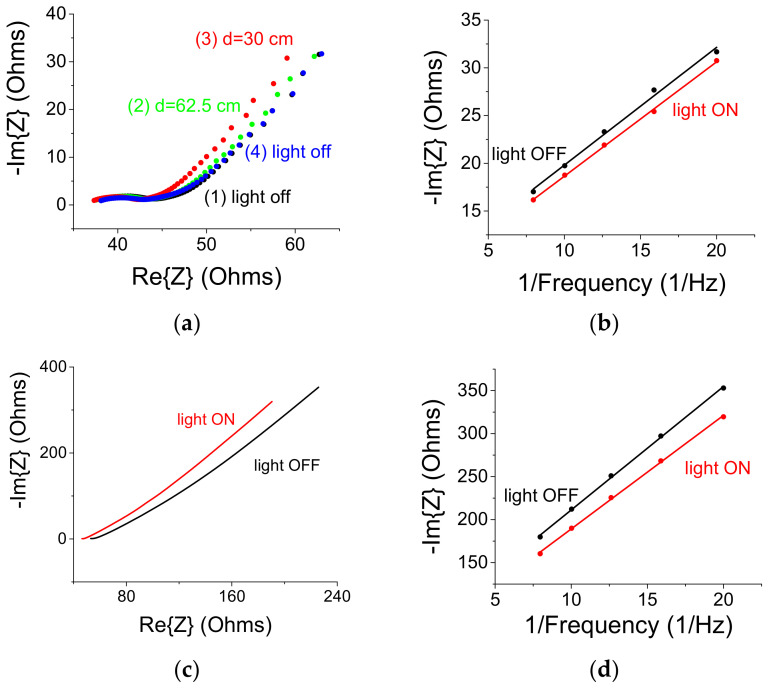
(**a**) An EIS Nyquist curve as a function of light. (1) White light OFF. (2) White light ON placed at distance d = 62.5 cm from the sample (translated to I = 3 mW/cm^2^ at the sample surface). (3) The white light was brought closer to the sample (d = 30 cm, I = 30 mW/cm^2^). (4) The white light source was turned OFF and its related curve follows case (1). (**b**) Plot of −Im{Z} vs. the inverse of the frequency exhibits a straight line with a slope proportional to the differential capacitance. (**c**) A sample with a high (imaginary) impedance exhibits a larger optical effect and its related differential capacitance curve is shown in (**d**). Note that under white light illumination, the slope of the curve, which is proportional to the inverse of the differential capacitance decreases.

Further experiments were carried out using Electrochemical Impedance Spectroscopy [24] (EIS, Metrohm, Herisau, Switzerland). The frequency range was 50 mHz to 50,000 Hz and the amplitude of the modulating signal was 20 mV. As shown in Figure 5a,c, the white light illumination affects the electrode’s impedance, an effect that amounted to 2%. A larger effect is noted for the (middle) diffusion region which became more capacitive under illumination. Lastly, the differential capacitance exhibits a constant value in the frequency region between 50 to 125 mHz exhibiting a small increase under illumination (Figure 5b,d). We note that a large double-layer capacitor exhibits smaller slopes in Figure 5b,d.

### 3.3. Thermal Considerations

**Figure 6:**
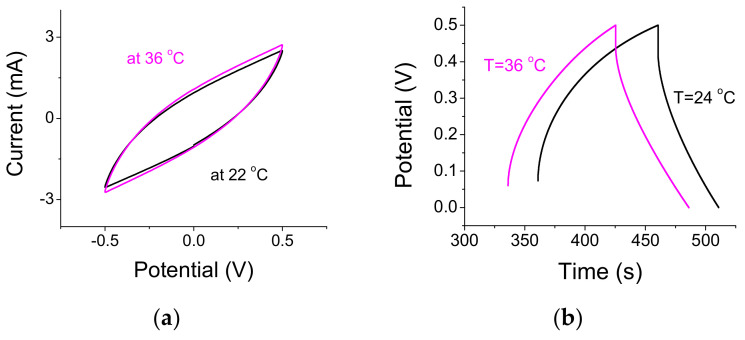

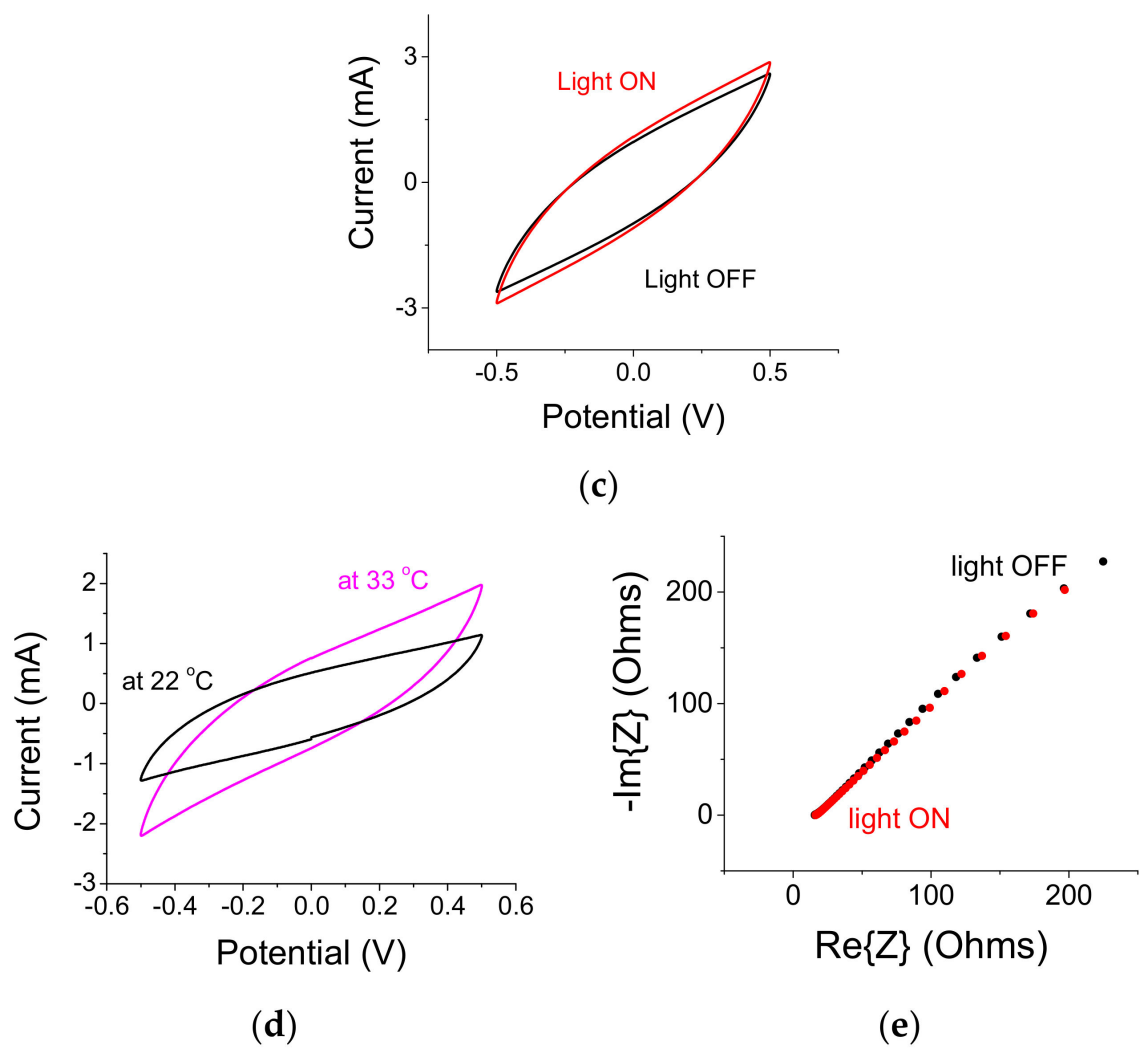
(**a**) The sample was heated when the light was OFF from 22 to 36 °C. CV curves were obtained at a scan of 0.1 V/s. (**b**) CD curves when a sample was measured at two stabilized temperatures. (**c**) CV curves for light OFF (room lighting) and light ON. (**d**) CV for A-C electrode embedded with CdSe/ZnS quantum dots. The relative capacitance change due to the thermal effect is ~35%. (**e**) EIS for A-C embedded with CdSe/ZnS quantum dots; the trend of the curve points to highly resistive samples at low frequencies.

The CV plot in Figure 6a has been obtained when collecting the CV data while continuously heating the sample from 22 °C to 36 °C at the rate of 0.1 °C/s. Only the curves in the beginning and at the end of the scan are shown. The light was turned OFF. Note the slight rotation of the curve as the sample heats up; its waist at zero potential has changed a bit too. The relative change from the first scan at 22 °C to the last at 36 °C was <10%. Results for CD experiments indicate a larger thermal effect of <20% (Figure 6b). Unlike CV experiments, here data were obtained with a hot plate at well-stabilized temperatures of 24 and 36 °C, respectively. Overall, the capacitance increase due to thermal effects is much larger than the thermal effect exhibited by n-Si embedded A-C [1]. The thermal effect with SiC particles may be attributed to the higher thermal conductivity of SiC in comparison with n-Si [25,26]. In Figure 6c, we show two CV curves for light OFF and light ON. Upon illumination, the temperature at the sample surface has elevated to 36 °C. The ‘as-is’ relative capacitance change was 12% (without including the fact that the exposed area was half of the entire area of the S-C). It was 24% when normalizing by the smaller exposed area. On the other hand, while the trend (see also Figure 3a) favors an optically related capacitance increase, one ought to acknowledge that the thermal and the optical effects are comparable and we cannot rule out the possibility of a heating effect by the optical source.

A very large thermal effect is obtained when the A-C electrodes are embedded with CdSe/ZnS quantum dots (Figure 6d). The relative capacitance change due to the thermal effect is ~35% compared to ~10% when the white light is turned ON and OFF (not shown). While most samples (A-C embedded with SiC, or A-C embedded n-Si) reached ~36 °C after a prolonged illumination, the unusual thermal effect in CdSe/ZnS embedded A-C electrode points to morphological changes, perhaps in the ligand that coats the dots. EIS for A-C embedded with CdSe/ZnS quantum dots exhibit a higher resistance at low frequencies rather than a higher capacitance trend (Figure 6e).

### 3.4. Model

A model was constructed to aid the understanding of these results. A small parallel plate capacitor filled with electrolyte was interfaced with two graphite-like electrodes. The electrodes were deposited on current collectors (terminals). The graphitic-like material was conductive; its dielectric constant was low, σ_r_ = 2, and its conductivity was modest, =10^5^ S/m. A charged particle (loosely defined here as QD) of radius 5 nm or 2.5 nm was placed inside the graphitic electrode. A CAD tool (Comsol, Stockholm, Sweden) was used to determine the potential distribution and the increased capacitance when the terminals were biased by ±1 V. In one case, the particle under illumination was modeled as a dipole. The particle surface closer to the upper positive electrode was decorated with one charge type, say, negative, and the particle surface away from that electrode was decorated with the complimentary type, say positive. In the second case, an ionized particle was modeled as having one type of surface charge, either entirely negative or, entirely positive.

**Figure 7:**
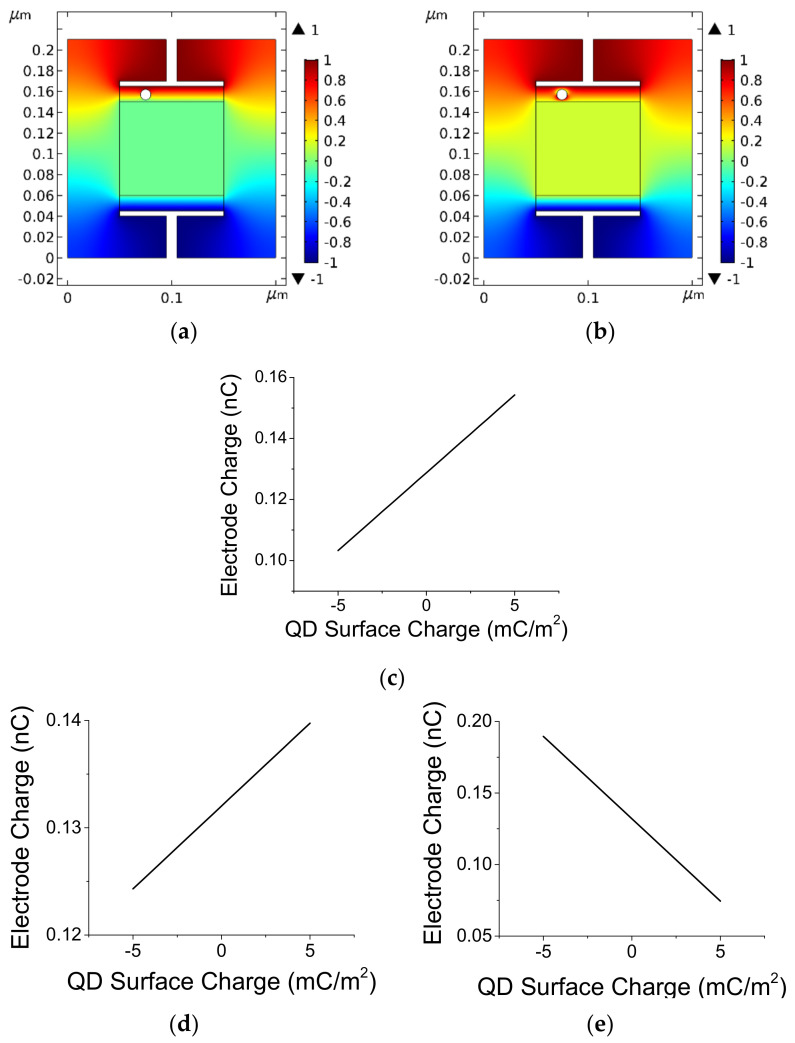
(**a**) Potential distribution when uncharged QD particle is embedded in a graphitic-like electrode. The cell is biased by ±1 V. (**b**) Potential distribution when the 5 nm particle acts like a dipole. (**c**) Change in the cell capacitance while varying the surface charge density of the dipole for an r = 5 nm particle. Positive surface charge values mean that the QD hemisphere that is away from the positive terminal is decorated with positive surface charges and the QD hemisphere that is closer to the positive terminal is decorated with negative surface charges. (**d**) Change in the cell capacitance while varying the surface charge density for an r = 2.5 nm dipole. Note the relatively large effect even though the overall charge has reduced by a factor of 2.5. (**e**) Change in the cell capacitance while varying the surface charge density of an ionized QD particle with r = 2.5 nm. Here, the charge type is covering the entire QD surface.

The potential distribution in the absence of light-induced charges is shown in Figure 7a. Note that the potential is constant within the electrolytic layer (the green-yellow region in the middle). The middle region is modeled as a floating potential region. Under illumination, free charges are excited when light is absorbed by the semiconductor particles. These free charges are displaced by the external bias of the capacitor (±1 V in our case). The excited (negative) electrons are attracted to the (upper) positive terminal, thereby attracting even more positive charges to the terminal in a self-promoting process. The extra charge to the terminal are provided by the biasing source. Such an effect increases the capacitance of the capacitor because the terminal charge is increased while its potential remains the same. Likewise, positive induced charges are attracted to a negatively biased terminal. Such a self-promoting process was used to increase the effective dielectric constant of materials, and the concept is known as artificial dielectrics, AD [27,28]. The AD concept has originally been devised for microwave and optical frequencies. Here the concept is adopted to very low frequencies and direct current (DC) cases. The introduction of a dipole at the electrode thus alters the potential distribution at the terminal (Figure 7b). Since the terminal voltage is kept constant, the cell capacitance is related to the charge that is accumulated on the terminal. When the light intensity increases, so will the induced charge on the particle surface, resulting in an increase of the cell capacitance (Figure 7c,d). The charge type convention for Figure 7c,d, is that a positive QD surface charge is decorating the QD hemisphere away from the positive terminal, as expected for a biased dipole Negative surface charge values are related to an atypical situation where the positive terminal attracts the positive QD charges, resulting in smaller capacitance values.

A particle is ionized when electrons are donated to the A-C electrode. The remaining positive charge increases the capacitance if the nearby terminal is negatively biased and decreases the cell capacitance if this terminal is positively biased (Figure 7e). An ionized particle, with some of its electrons stripped away, would impact the cell capacitance in an asymmetrical fashion (which we do not see in the experimental data).

As a note, a surface charge of 5 mC/m^2^ translates to ~6.25 and 2.5 electrons per particle of radii r = 5 nm and r = 2.5 nm, respectively.

## 4. Conclusions

Supercapacitors whose A-C electrodes were embedded with submicron SiC particles have shown a substantial capacitance increase: the optically-related capacitance increase may be as large as ~34% (68% when the illuminated area is taken into account). Thermal effects amount to <20% of the overall relative change using CD experiments. The capacitance increase is attributed to optically-induced polarization of the semiconductor particles. When comparing dry and wet samples, the relative absorption indicates an unusual scattering mechanism that cannot be accounted for by just the absorbing water. Finally, a large anomaly is reported when CdSe/ZnS QD are embedded in A-C electrodes; there the thermal effect is much larger than the optical one. Further understanding of these phenomena, extending this study to other semiconductor particles, understanding the role of electrolytes in the optical scattering mechanism may help us design better optically controlled S-C elements.

## Data Availability

Data Available upon reasonable request.
